# Mucosal Autoimmunity to Cell-Bound GP2 Isoforms Is a Sensitive Marker in PSC and Associated With the Clinical Phenotype

**DOI:** 10.3389/fimmu.2018.01959

**Published:** 2018-08-28

**Authors:** Mandy Sowa, Rafał Kolenda, Daniel C. Baumgart, Johann Pratschke, Maria Papp, Tamas Tornai, Jaroslaw Suchanski, Dimitrios P. Bogdanos, Maria G. Mytilinaiou, Jutta Hammermann, Martin W. Laass, Karsten Conrad, Christoph Schramm, Andre Franke, Dirk Roggenbuck, Peter Schierack

**Affiliations:** ^1^Institute of Biotechnology, Faculty Environment and Natural Sciences, Brandenburg University of Technology Cottbus–Senftenberg, Senftenberg, Germany; ^2^Department of Biochemistry and Molecular Biology, Faculty of Veterinary Medicine, University of Environmental and Life Sciences, Wroclaw, Poland; ^3^Inflammatory Bowel Disease Center, Department of Gastroenterology and Hepatology, Charité Medical School, Humboldt-University of Berlin, Berlin, Germany; ^4^Department of Surgery, Charité Medical School, Humboldt-University of Berlin, Berlin, Germany; ^5^Department of Internal Medicine, Division of Gastroenterology, Faculty of Medicine, University of Debrecen, Debrecen, Hungary; ^6^Division of Transplantation Immunology and Mucosal Biology, King's College London School of Medicine at King‘s College Hospital, London, United Kingdom; ^7^Department of Rheumatology and Clinical Immunology, School of Health Sciences, University of Thessaly, Larissa, Greece; ^8^Children's Hospital, Medical Faculty Carl Gustav Carus, Technical University Dresden, Dresden, Germany; ^9^Institute of Immunology, Technical University Dresden, Dresden, Germany; ^10^I. Department of Medicine and Martin Zeitz Centre for Rare Diseases, University Medical Centre Hamburg-Eppendorf, Hamburg, Germany; ^11^Institute of Clinical Molecular Biology, Christian-Albrechts-University, Kiel, Germany; ^12^GA Generic Assays GmbH, Berlin, Germany

**Keywords:** zymogen granule glycoprotein 2, primary sclerosing cholangitis, cirrhosis, cholangiocarcinoma, immunoglobulin A

## Abstract

**Introduction:** Zymogen granule glycoprotein 2 (GP2) was demonstrated as first autoimmune mucosal target in primary sclerosing cholangitis (PSC) associated with disease severity. Autoantibodies to four GP2 isoforms (aGP2_1−4_) were found in patients with inflammatory bowel diseases but reactivity against specific GP2 epitopes has not been investigated in PSC yet. Hence, the prevalence of aGP2_1−4_ and their association with the PSC phenotype for risk prediction were examined.

**Methods:** GP2 isoforms were stably expressed as glycosylphosphatidyl - inositol-anchored molecules in the membrane of HEp-2 cells and used as autoantigenic targets in indirect immunofluorescence assay (IFA). aGP2_1−4_ IgA and IgG were detected by IFA in 212 PSC patients of four European university hospitals and 145 controls comprising 95 patients with cystic fibrosis and 50 healthy subjects.

**Results:** Combined aGP2_1_ and aGP2_4_ IgA testing with a sensitivity of 66.0% and a specificity of 97.9% resulted in the best diagnostic performance (Youden index: 0.64) regarding all aGP2 and combinations thereof. aGP2_4_ IgA positivity is significantly associated with the presence of cirrhosis in PSC (*p* = 0.0056). Logistic regression revealed the occurrence of aGP2_1_ IgA (odds ratio [OR] 1.38, 95% confidence interval [CI]: 1.03–1.86) and aGP2_4_ IgA (OR 1.52, 95%CI: 1.07–2.15) along with male gender (OR 0.51, 95%CI: 0.27–0.97) and older age (OR 1.03 95%CI: 1.01–1.05) as significant risks for the concomitant presence of cirrhosis in PSC.

**Conclusions:** Combined aGP2_1_ and aGP2_4_ IgA analysis is preferred to single aGP2 isoform analysis for sensitive PSC autoantibody testing. Positivity for aGP2_1_ and aGP2_4_ IgA is associated with cirrhosis in PSC and could be used for risk stratification.

## Introduction

Primary sclerosing cholangitis (PSC), a chronic immune-mediated, life threatening, genetically predisposed, cholestatic liver illness, is associated with the co-occurrence of inflammatory bowel disease (IBD) and in particular with the phenotype thereof (1, 2). The prevalence of PSC is estimated at up to 16.2 per 100,000 individuals and still rising (3, 4). There is a clinical need for markers predicting PSC severity and prognosis despite recent progress regarding the use of certain serum and bile proteins (5, 6). Further, IgA to zymogen granule glycoprotein 2 (GP2) was identified as a novel marker candidate for disease severity and cholangiocarcinoma in PSC (7, 8). Glycoprotein 2 is a microbe-sensing (9, 10) and immunomodulating molecule (11) with two major sources (pancreas and intestine) (12). Of note, GP2 was originally identified as a target of Crohn's disease (CD)-specific pancreatic antibodies (13, 14). Upon specific binding to FimH, GP2 interacts selectively with bacterial species including pathogens (10) and, thus, may determine both innate and acquired immune responses to the intestinal microbiota (11, 15). There is mounting evidence that mucosal interactions between the intestinal microbiota and host immune responses partake in the development of chronic inflammatory disorder of the gastrointestinal tract (12, 16–18).

Since the first report of GP2's over-expression in the inflamed intestine of CD patients (13), several cross-sectional and prospective studies demonstrated the association of autoantibodies (autoAbs) to GP2 (aGP2) with the stricturing and/or stenosing CD phenotype and disease severity (18, 19), earlier surgical recurrence after first surgery (20), as well as de-novo development of CD in patients with suspected ulcerative colitis (UC) after ileal pouch surgery and development of subsequent pouchitis (21). Thus, autoimmunity to GP2 appears to be a stratification factor of the clinical phenotype in IBD (18).

Given the close association of PSC with IBD, the occurrence of aGP2 IgA in severe PSC (7) provided further evidence for the correlation of the mucosal loss of tolerance to GP2 with fibrostenotic changes as reported in IBD (22). Remarkably, a recent comprehensive retrospective outcome analysis of 7,121 PSC patients at 37 centers in Europe, North America, and Australia revealed that 70% of them developed IBD at some point (1). Conversely, PSC appeared to be underestimated around three-fold in long-term IBD and to progress in subclinical IBD patients (23).

In total, four human GP2 isoforms (GP2_1−4_) were identified (18, 24) and respective autoAbs detected in patients with IBD which demonstrated differing test performances by enzyme-linked immunosorbent assay (ELISA) (20, 25, 26). Hence, stable HEp-2 cell-lines expressing GP2 isoforms as glycosylphosphatidylinositol (GPI)-anchored membrane molecules and one cell line with an empty vector as control were generated to elucidate the role of loss of tolerance to GP2 isoforms in PSC. Consequently, IgG and IgA aGP2_1−4_ by indirect immunofluorescence assay (IFA) in patients with PSC and controls were determined.

## Methods

### Patients

Patients with PSC were recruited from four European university hospitals specialized in autoimmune liver diseases (Table 1). All PSC patients were examined clinically and endoscopically for concomitant IBD and autoimmune hepatitis (AIH). The diagnosis of PSC and IBD was based on clinical, radiologic, endoscopic, and histologic evaluation (27, 28, 29).

**Table 1:** Demographic and clinical data of patients and controls.

	***n***	**Age (IQR)**	***f* (%)**	**IBD (%)**	**CD (%)**	**UC (%)**	**AIH (%)**	**Cirrhosis (%)**	**CCa (%)**	**LTx (%)**
PSC	212	43.0 (23.3)	70 (33.0)	136 (64.2)	17 (8.0)	119 (56.1)	20 (9.4)	86 (31.6)	5* (3.7)	81 (38.2)
Berlin	23	52.5 (17.5)	6 (26.1)	19 (82.6)^§3^	2 (8.7)	17 (73.9)	1 (4.3)	19 (82.6)	0	19 (82.6)
Hamburg	30	50.0 (17.3)	18 (60.0)^§2^	15 (50.0)	4 (13.3)	11 (36.7)^§5^	5 (16.7)	3 (10.0)^§6^	0	0
London	83	46.3 (18.7)	23 (27.7)	53 (63.9)	1 (1.2)^§4^	52 (62.7)	5 (6.0)	49 (59.0)^§8^	5 (6.0)	57 (68.8)
Debrecen	76	34.1 (21.6)^§1^	23 (30.3)	49 (64.5)	10 (13.2)	39 (51.3)	9 (11.8)	15 (19.7)^§7^	0	6 (7.9)^§9^
Controls	145	26.9 (22.1)^&^	63 (43.4)	0^$^	0^$^	0^$^	0^$^	0^$^	0	0
CF	95	15.6 (20.9)^&^	44 (46.3)^$^	0^$^	0^$^	0^$^	0^$^	0^$^	0	0
HS	50	36.0 (18.0)	19 (38.0)	0^$^	0^$^	0^$^	0^$^	0^$^	0	0

AIH, autoimmune hepatitis; CCa, cholangiocarcinoma; CD, Crohn's disease; CF, cystic fibrosis; f, females; HS, healthy subjects; IQR, interquartile range; LTx, liver transplantation; n, number; PSC, primary sclerosing cholangitis; UC ulcerative colitis.

* *Related to 136 patients with PSC*.

Comparison of the prevalence in all patients with primary sclerosing cholangitis with control groups:

& *p < 0.05 by Kruskal-Wallis test for continuous variables*.

Comparison of the prevalence in all patients with primary sclerosing cholangitis with control groups:

$ *p < 0.05 by Fisher's exact test for dichotomous values*.

Comparison of the prevalence within the cohorts of patients with primary sclerosing cholangitis:

§1 *Debrecen vs. Berlin, Hamburg and London, respectively (p < 0.05 by Kruskal–Wallis test)*.

§2 *Hamburg vs. Berlin, Debrecen and London, respectively (p < 0.05 by Fisher's exact test)*.

§3 *Berlin vs. Hamburg (p < 0.05 by Fisher's exact test)*.

§4 *London vs. Hamburg and Debrecen, respectively (p < 0.05 by Fisher's exact test)*.

§5 *Berlin vs. Hamburg and London, respectively (p < 0.05 by Fisher's exact test)*.

§6 *Hamburg vs. Berlin and London, respectively (p < 0.05 by Fisher's exact test)*.

§7 *Debrecen vs. Berlin and London, respectively (p < 0.05 by Fisher's exact test)*.

§8 *London vs. Berlin (p < 0.05 by Fisher's exact test)*.

§9 *Debrecen vs. Berlin and London, respectively (p < 0.05 by Fisher's exact test)*.

In total, 145 gender-matched controls were enrolled in this study comprising 95 patients with cystic fibrosis (CF) and 50 healthy subjects (HS). The patients with CF, a multi-systemic disorder with exocrine pancreatic insufficiency and biliary cirrhosis with a different pathogenesis, were included as disease controls with regard to PSC. The 50 apparently healthy subjects (HS) with no liver or intestinal pathology were both age- and gender-matched.

The study was approved by the ethics committees of the participating centers. Written informed consent was obtained from all patients included in this study. The study was conducted in accordance with the principles of the Declaration of Helsinki (World Medical Association Declaration of Helsinki 1989).

### Generation of GP2-expressing cell lines

Stable HEp-2 cell lines expressing membrane GPI-anchored GP2 isoforms were generated through transduction with lentiviruses. Briefly, coding sequences of GP2 isoforms sequences were amplified with PCR and cloned into pLVX-IRES-puro plasmids each using T4 DNA ligase (ThermoFisher Scientific, Waltham, USA) in accordance with the manufacturer's protocol. To confirm successful cloning, plasmids were Sanger sequenced. For transduction, lentiviruses were produced using the Lenti-X Lentiviral Expression System (Clontech Laboratories, Mountain View, USA). Thus, an 80% confluent Lenti-X 293T cell line was co-transfected with the six plasmids containing GP2 isoforms or an “empty” vector. The harvested supernatants were concentrated by centrifugation in Amicon Ultra-15 centrifugal filter units (Merck Millipore, Darmstadt, Germany) and employed for transduction of HEp-2 cells. Selection of successfully transduced cells was performed by adding the antibiotic puromycin to cell culture.

Confirmation of GP2 transduction into HEp-2 cells was done with reverse transcription quantitative polymerase chain reaction (RT qPCR). In brief, RNA from transduced cells was isolated with RNeasy Mini Kit according to the manufacturer's protocol (Qiagen, Hilden, Germany). After RT reaction using Maxima First Strand Synthesis Kit (ThermoFisher Scientific), qPCR was performed in the CFX96 Touch Real-Time PCR Detection System (Bio-Rad Laboratories, München, Germany) with following primers: 5- ATCAACGTGATTCCACCATCC-3 and 5- TTGAGCAAGAAGGCTGGC-3 (for GP2 gene); 5-AAATGTTTCATTGTGGGAGC-3 and 5- ATATGAGGCAGCAGTTTCTC-3 (for RPLP0 gene). RPLP0 was used as reference gene. Obtained PCR products were analyzed by electrophoresis.

Expression of GP2 isoform proteins was confirmed by Western blotting. Briefly, transduced HEp-2 cells were lysed and the lysate run on SDS-PAGE. Separated bands transferred on the blotting membrane were developed with a rabbit polyclonal antibody to GP2 reactive with all isoforms (GA Generic Assays, Dahlewitz, Germany). Isoforms of GP2 were revealed by a secondary anti-rabbit antibody conjugated with horse radish peroxidase employing enhanced chemiluminescence.

Membrane expression of GPI-anchored GP2 isoforms was confirmed by flow cytometry analysis.

### Detection of IgG and IgA to GP2 isoforms

IgG and IgA to GP2 isoforms were determined by IFA employing stably transduced HEp-2 cells expressing membrane GPI-anchored GP2 isoforms 1-4. Briefly, cells were fixed on glass slides as described elsewhere (30) and incubated with 1 in 20 diluted sera for 1 h at room temperature. HEp-2 cells transduced with an empty vector were used as negative control. After washing, bound autoAbs to GP2 isoforms were revealed by incubation of polyclonal anti-human IgG or IgA antibodies conjugated to fluorescein isothiocyanate (FITC) (Agilent, Santa Clar, USA) for 1 h at room temperature. A fluorescent microscope (Axiovert 40, Zeiss, Göttingen, Germany) was used to read specific staining of HEp-2 cells. Brighter fluorescent staining of the cellular membrane of transduced HEp-2 cells in comparison with HEp-2 cells transduced with an empty vector was scored positive.

### Statistical methods

Data were tested for normality by the Kolmogorov-Smirnov-test and non-normally distributed data were reported by median and quartile ranges. The two-tailed, Mann-Whitney and Kruskal–Wallis tests were used to test for statistically significant differences of independent samples in 2 and more groups, respectively. Prevalence comparison between groups was performed by two-tailed Fisher's exact test. Logistic regression analysis was employed to test for the influence of explanatory (independent) variables on a binomial response variable and to detect possible clinical confounders on such association (age, gender, concomitant IBD, concomitant overlap with AIH) by a backward exclusion strategy resulting in adjusted odds ratios. *P* < 0.05 was considered as significant. MedCalc software version 12.7.0.0 (MedCalc, Mariakerke, Belgium) was used for performing statistical analysis.

## Results

### Detection of autoantibodies to GP2 isoforms by indirect immunofluorescence

**Figure 1:**
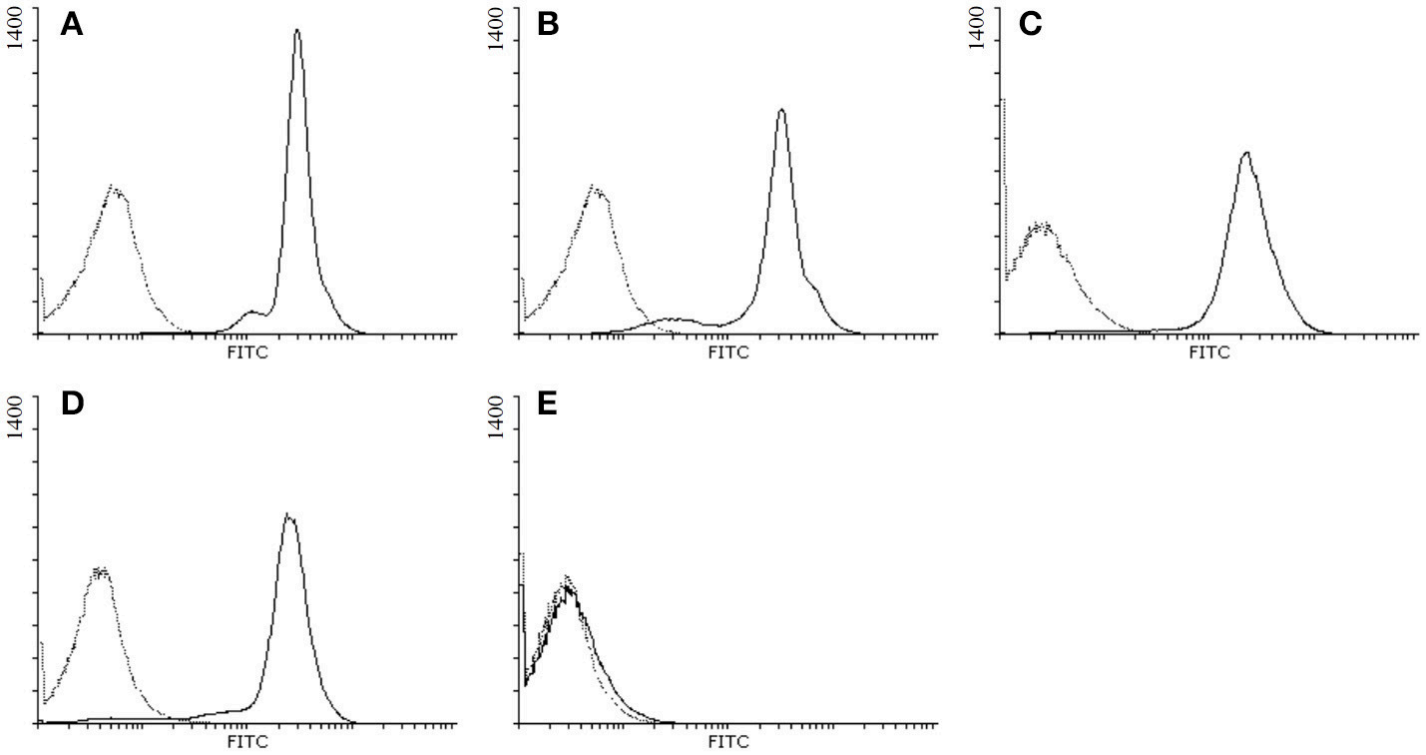
Detection of the membrane expression of GP2 isoforms in HEp-2 cells by flow cytometry. GP2 expressed in HEp-2 cells was stained with polyclonal antibodies raised against full length human GP2 followed by FITC-conjugated anti-rabbit IgG: **(A)** HEp-2 cells expressing human GP2 isoform 1; **(B)** GP2 isoform 2; **(C)** GP2 isoform 3; **(D)** GP2 isoform 4; **(E)** HEp-2 cells transduced with an empty vector; black solid lines: primary and secondary antibody staining; black dotted lines: secondary antibody staining only.

GP2 isoforms were expressed stably in HEp-2 cells as GPI-anchored molecules in the membrane of these cells by lentiviruses transduction. As control, one cell line was transduced with an empty vector only. The presence of membrane-bound GP2_1_ to GP2_4_ in the respective lines and their absence in the empty vector cell line was confirmed by FACS analysis (Figure 1).

**Figure 2:**
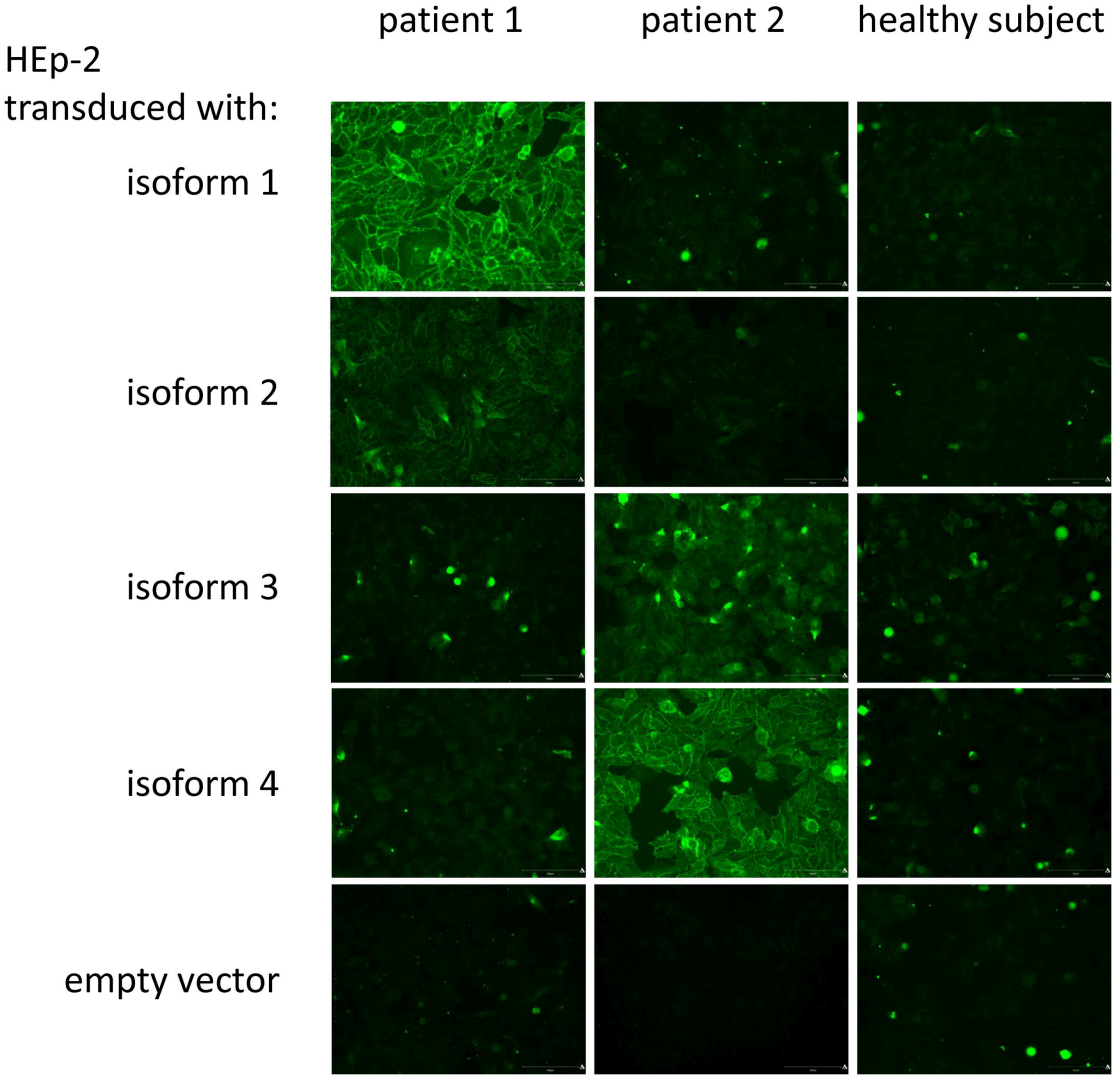
Indirect immunofluorescence assay for the detection of IgA to GP2 isoforms: Exemplarily, two patient sera and one serum of a healthy subject as control were run on HEp-2 cells transduced with GP2 isoforms 1 (GP2_1_) to 4 (GP2_4_) with glycosylphosphatidylinositol anchor and an empty vector, respectively. Patient 1 demonstrated a strong specific binding to membrane-bound GP2_1_ and a weak one to GP2_2_, whereas patient 2 showed the typical binding pattern for a strong positive binding to GP2_4_ and a weak one for GP2_3_. The healthy subject did not reveal a positive membrane-reactive pattern on the respective transduced HEp-2 cells.

For the detection of aGP2_1_ to aGP2_4_ by IFA, cells of each line were fixed to conventional glass slides and used as targets for specific autoAb analysis (Figure 2)_._

### Occurrence of IgA and IgG to GP2 isoforms in patients and controls

IgA and IgG against GP2_1−4_ were determined in 212 patients with PSC of four European hospitals and 145 gender-matched controls. Of note, the 50 HS included as controls were gender- as well as aged-matched to all PSC patients (Table 1). Patients with PSC of the Debrecen cohort were significantly younger compared to the remaining three PSC cohorts whereas the Hamburg cohort had a significantly higher median age (*p* < 0.05, respectively).

Apart from aGP2_3_, all other aGP2 demonstrated significantly elevated prevalences in PSC patients compared with controls including HS and patients with CF (*p* < 0.05, respectively) (Table 2). However, this did not hold true for all PSC cohorts of the four different centers.

**Table 2:** Frequency of IgA and IgG against GP2 isoforms 1 (aGP2_1_) to 4 (aGP2_4_) detected by indirect immunofluorescence assay on stabile isoform-transduced HEp2 cells in 212 patients with primary sclerosing cholangitis (PSC) from different hospitals and 145 controls.

		**aGP2**_**1**_		**aGP2**_**2**_		**aGP2**_**3**_		**aGP2**_**4**_		**aGP2**_**1/2/3/4**_		**aGP2**_**1/4**_	
	***n***	**IgA (%)**	**IgG (%)**	**IgA (%)**	**IgG (%)**	**IgA (%)**	**IgG (%)**	**IgA (%)**	**IgG (%)**	**IgA (%)**	**IgG (%)**	**IgA and/ or IgG (%)**	**IgA (%)**
PSC	212	100 (47.2)**	92 (43.4)**	21 (9.9)*	40 (18.9)*	10 (4.7)	31 (14.6)	103 (48.6)**	81 (38.2)**	140 (66.0)**	119 (56.1)**	154 (72.6)**	140 (66.0)**
Berlin	23	6 (26.1)**	7 (30.4)*	0	0	0	0	3 (13.0)*	2 (8.7)	8 (34.8)**	8 (34.8)	10 (43.5)	8 (34.8)**
Hamburg	30	11 (36.7)**	12 (40.0)*	5 (16.7)*	1 (3.3)	1 (3.3)	1 (3.3)	9 (30.0)**	8 (26.7)*	16 (53.3)**	16 (53.3)*	17 (56.7)*	16 (53.3)**
London	83	42 (50.6)**	39 (47.0)**	2 (2.4)	28 (33.7)**	4 (4.8)	26 (31.3)*	52 (62.7)**	40 (48.2)**	59 (71.1)**	50 (60.2)**	68 (81.9)**	59 (71.1)**
Debrecen	76	41 (53.9)**	34 (44.7)**	14 (18.4)**	11 (14.5)	5 (6.6)	4 (5.3)	39 (51.3)**	31 (40.8)**	57 (75.0)**	45 (59.2)**	59 (77.6)**	57 (75.0)**
Controls	145	2 (1.4)	11 (7.6)	1 (0.7)	12 (8.3)	4 (2.8)	20 (13.8)	1 (0.7)	11 (7.6)	6 (4.1)	33 (22.8)	34 (23.4)	3 (2.1)
CF	95	1 (1.1)	8 (8.4)	1 (1.1)	9 (9.5)	4 (4.2)	18 (18.9)^$^	1 (1.1)	9 (9.5)	5 (5.3)	26 (27.4)^$^	26 (27.4)	2 (2.1)
HS	50	1 (2.0)	3 (6.0)	0	3 (6.0)	0	2 (4.0)	0	2 (4.0)	1 (2.0)	7 (14.0)	8 (16.0)	1 (2.0)
YI (PSC vs. controls)		0.46	0.36	0.09	0.11	0.02	0.00	0.48	0.31	0.62	0.33	0.49	0.64

CF, cystic fibrosis, f, females; HS, healthy subjects; IQR, interquartile range; n, number; YI, Youden index (specificity+sensitivity-1)

Comparison of the prevalence of positive aGP2 in patients with primary sclerosing cholangitis with controls (n = 145):

* p < 0.05,

** p < 0.0001, by Fisher's exact test, respectively.

Comparison of the prevalence of positive aGP2 in patients with cystic fibrosis with healthy subjects (n = 50):

$ *p < 0.05 by Fisher's exact test*.

Regarding IgA reactivity, aGP2_1_ (47.2%) and aGP2_4_ positivity (48.6%) revealed the highest frequencies in PSC patients resulting in an even significantly elevated combined positive rate of 66.0% (aGP2_1and/or4_ IgA) compared with both rates of single aGP2 isoform IgA testing (*p* < 0.0001, = 0.0004, respectively). Analysis of all four aGP2 isoform IgA did not increase the positive rate further. Apart from aGP2_3_ IgA, all other aGP2 isoform IgA demonstrated significantly lower prevalences in controls. Thus, aGP2_1and/or4_ IgA testing revealed the best Youden index (YI) of 0.64 being a measure of assay performance.

In terms of IgG, aGP2_1_, and aGP2_4_ testing revealed the highest positive rates in PSC patients, too. However, their prevalences were lower in contrast to the corresponding IgA, but only the difference for aGP2_4_ reached significance (*p* = 0.0395). Further, both aGP2 isoform IgG had significantly more positives in the control groups (*p* < 0.5, respectively). That resulted in halved YIs for both and, thus, demonstrated a poorer assay performance for the discrimination of patients with PSC from controls in comparison to corresponding IgA analysis.

### Association of IgA and IgG to GP2 isoforms with PSC phenotypes

The possible association of the presence of IgA and IgG to GP2_1−4_ in PSC patients with performed liver transplantation (LTx) and concomitant occurrence of autoimmune hepatitis, cirrhosis; cholangiocarcinoma, CD, UC, IBD (CD or UC) was investigated by Fisher's exact test (Table 3). Further, established associations were investigated by logistic regression analysis to analyze the influence of confounding factors.

**Table 3:** Positive and negative (italic) significant associations of IgA and IgG against GP2 isoforms 1 (aGP2_1_) to 4 (aGP2_4_) with the clinical phenotype in 212 patients with primary sclerosing cholangitis (PSC) by Fisher's exact test.

	**N**	**IBD**	**CD**	**UC**	**AIH**	**Cirrhosis**	**CCa**	**LTx**
PSC	212		*aGP2_1/4_ IgA* *p = 0.01386 aGP2_1/2/3/4_ IgA* *p = 0.0076*			aGP2_4_ IgA p = 0.0051 aGP2_1/4_ IgA p = 0.0056 aGP2_2_ IgG p = 0.0199 aGP2_4_ IgG p = 0.0447	aGP2_3_ IgG p < 0.0001	*aGP2_2_ IgA* *p = 0.0006*
Berlin	23							
Hamburg	30			*aGP2_2_ IgA* *p = 0.0472*				
London	83	*aGP2_3_ IgG* *p = 0.0288*				aGP2_4_ IgA p = 0.0055 aGP2_1/2/3/4_ IgA p = 0.0144	aGP2_3_ IgG p = 0.0316	
Debrecen	76					aGP2_2_ IgA p = 0.0261 aGP2_2_ IgG p = 0.0349		

*AIH, autoimmune hepatitis; CCa, cholangiocarcinoma; CD, Crohn's disease; LTx, liver transplantation; n, number; PSC, primary sclerosing cholangitis; UC ulcerative colitis*.

#### Association of IgA and IgG to GP2 isoforms with cirrhosis

A significantly positive association of aGP2 isoform IgA and IgG positivity in PSC was established for the concomitant occurrence of cirrhosis. Thus, aGP2_1_ and aGP2_4_ IgA as well as aGP2_2_ and aGP2_4_ IgG were more prevalent in PSC patients with cirrhosis than in those without (*p* < 0.05, respectively). Similar positive associations could be found in the larger cohorts from London and Debrecen, too. Logistic regression analysis for the risk analysis of the occurrence of cirrhosis in all 212 PSC patients confirmed aGP2_1_ and aGP2_4_ IgA as independent predictors in older male PSC patients (Table 4).

**Table 4:** Logistic regression analysis of independent variables for the risk prediction of liver transplantation (LTx) and the occurrence of cirrhosis in 212 patients with primary sclerosing cholangitis (PSC).

**Dependent variable**	**Independent variable**	**Coefficient**	**SE**	**OR**	**95% CI**	***P***
cirrhosis	aGP2_1_ IgA	0.3243	0.1514	1.3831	1.0279–1.8609	0.0322
	aGP2_4_ IgA	0.1729	0.1771	1.5178	1.0727–2.1478	0.0185
	age	0.0273	0.0100	1.0277	1.0077–1.0480	0.0063
	gender	−0.6693	0.3235	0.5121	0.2716–0.9654	0.0385
LTx	aGP2_2_ IgA	−3.02104	1.27092	0.0488	0.0040–0.5886	0.0175
	cirrhosis	3.70772	0.49727	40.7608	15.3800–108.0262	< 0.0001
	AIH overlap	−2.63423	1.00286	0.0718	0.0101–0.5124	0.0086
	UC	1.82313	0.49012	6.1912	2.3691–16.1799	0.0002

*The presence of IgA and IgG to GP2 isoforms 1 (aGP2_1_) to 4 (aGP2_4_) and the concomitant occurrence of inflammatory bowel disease, Crohn's disease, ulcerative colitis (UC), and autoimmune hepatitis overlap as well as age and gender were used as possible predictive independent variables for the logistic regression analysis*.

*CI, confidence interval, OR, adjusted odds ratio; SE, standard error*.

#### Association of aGP2_2_ IgG with PSC patients without LTx

A significantly negative association of aGP2_2_ IgA was revealed for LTx performed in PSC patients of all cohorts. This significantly more prevalent aGP2_2_ IgA occurrence in PSC patients without LTx was not detected in the single PSC cohorts but confirmed by logistic regression analysis as independent predictor in PCS patients without LTx demonstrating concomitant cirrhosis, UC and no AIH overlap (Table 4).

#### Association of IgA and IgG to GP2 isoforms with IBD

Patients with PSC and concomitant CD demonstrated a significantly lower prevalence of aGP2_1/4_ and aGP2_1/2/3/4_ IgA. However, this association was neither found in the single PSC cohorts nor confirmed by logistic regression analysis. There was no further association of all PSC patients with and without IBD, UC or CD regarding the concomitant presence of aGP2 isoform autoAbs by Fisher's exact test. Only the London cohort demonstrated significantly less frequent aGP2_3_ IgG in PSC patients with concomitant IBD and the Hamburg cohort significantly less frequent aGP2_2_ IgA in patients with UC (*p* < 0.05, respectively) (Table 3).

Logistic regression analysis revealed the concomitant occurrence of UC as an independent risk factor for liver transplantation along with cirrhosis. The presence of GP2_2_ IgA and AIH overlap were negative predictors in this regard (Table 4).

## Discussion

The recent reports of the occurrence of aGP2 IgA in patients with large biliary duct disorders including PSC and of aGP2 IgA as marker of severe disease, as well as cholangiocarcinoma ushered in a new era in PSC serology (7, 31). Until recently, perinuclear antineutrophil cytoplasmic antibodies (ANCA) have been considered as main serological marker of PSC (32) and particularly ANCA IgA has been associated with cirrhosis linked to intestinal infections (33). However, attempts to identify the respective autoantigenic ANCA as well as other PSC-specific targets have been inconclusive so far (34). Conversely, IgG to proteinase 3 (PR3) being a cytoplasmic ANCA target could be detected by highly sensitive enzyme immunoassays or microbead-based immunoassays in up to 44% of patients with PSC (35, 36). Thus, apart from the neutrophilic target PR3, GP2 was identified as an autoantigenic target in PSC and questions on a possible pathogenic role of respective specific IgA being the mucosal immunoglobulin in contrast to IgG were raised. Since four GP2 isoforms were discovered and specific IgG and IgA against them were described in patients with IBD (24–26, 37), this study attempted to ascertain the frequency of IgG and IgA to GP2 isoforms in PSC and their possible relation to the PSC phenotype as stratification factor in PSC.

In contrast to recent reports demonstrating preferentially aGP2 IgA in PSC (7, 8), this study revealed both IgA as well as IgG against GP2 isoforms.

The IgG to GP2 isoforms determined in this study demonstrated both lower sensitivities and specificities in contrast to IgA against the corresponding GP2 isoforms which resulted in poorer assay performances of the former. Of note, apart from significantly higher positive rates of IgG to GP2 isoforms in PSC, we detected a significantly elevated prevalence of IgG GP2_3_Ab in control patients with CF (18.9%) vs. healthy controls (4.0%). This finding warrants further investigation and could indicate a different loss of tolerance to GP2 isoforms in CF.

In terms of discrimination of PSC from controls, aGP2_1_ IgA (47.2%) and aGP2_4_ IgA-positives (48.6%) revealed the highest frequencies amongst all aGP2. Interestingly, combination of both led to a significantly elevated sensitivity of 66.0%. This was a significantly higher prevalence than the corresponding total prevalence reported by Jendrek et al. (66.0 vs. 48.7%, *p* < 0.0001). Thus, combined testing of both aGP2_1_ and aGP2_4_ IgA appears more sensitive than the determination of IgA to just one GP2 isoform. Indeed, the single positivity rates of aGP2_1_ and aGP2_4_ IgA in this study were not significantly different from the rate reported elsewhere. Further, due to the higher specificity, combined aGP2_1_ and GP2_4_ IgA testing demonstrated the best diagnostic performance for PSC by a YI of 0.64 regarding the analysis of all aGP2 and combinations thereof.

As GP2_1_ and GP2_4_ represent long (537 amino acids) and short GP2 isoforms (387 amino acids), respectively, they could bear differing epitopes (24). Remarkably, GP2_2_ and GP2_3_ which differ from GP2_1_ and GP2_4_ in just three amino acids (valine-proline-arginine), respectively (25), demonstrated significantly diminished IgA binding in PSC. This adds support to the notion that GP2 isoforms bear different autoantigenic epitopes, all of which should be contained in GP2-antigenic preparations in aGP2 immunoassays for proper and accurate autoAb testing.

IgG and in particular IgA against GP2 isoforms revealed different associations with the PSC phenotype. In fact, aGP2_1_ and aGP2_4_ IgA were demonstrated as positive predictors of cirrhosis in older males with PSC. The cirrhosis in PSC is mainly characterized by extensive fibrosis around the larger bile ducts (4). An association of aGP2 IgG and IgA detected by ELISA with the stenosing/stricturing phenotype in patients with CD, which is characterized by fibrostenotic changes either, was established in one longitudinal and several cross-sectional studies (18). Further, the need for surgical resection and repeated surgical intervention in CD was associated with the occurrence of aGP2 (20, 22). Wölfel et al. demonstrated a significant association of aGP2_1_ IgA and a tendency for′ GP2_4_ IgA with earlier surgical recurrence in CD (20). Further, aGP2_1_ and aGP2_2_ IgA were reported to be significantly associated with the stenosing/stricturing and severity of disease but not with disease activity. Thus, IgA against GP2 isoforms might be a serological marker for the development of fibrostenotic changes in both the gut and larger bile ducts.

There is clearly a need for risk stratification in PSC (5, 38) and aGP2 IgA could be of prognostic value like autoAbs to gp210 do in primary biliary cholangitis (39). Given a potential ascending pathophysiology of PSC, aGP2_1/4_ IgA could be a stage-defining biomarker (40). This is of interest, as aGP2 IgA secreted onto mucosal surfaces might mediate the up-take of GP2-covered bacteria by GP2-bearing M cells of the intestinal follicle-associated epithelium (15, 41) and, thus, partake in the development of severe complications or even pre-tumor stages. Patients with PSC appear to demonstrate a gut microbial signature distinct from both normal individuals and patients with UC without liver disease whereas their microbial signature is not dependent on the occurrence of IBD (42). Thus, fecal microbiota profiles can be used as markers of PSC (43). Altogether, that hints at an involvement of the microbiota in the pathophysiology of PSC which was demonstrated in a well-established mouse models for PSC and spontaneous bile duct inflammation (44, 45).

Interestingly, CD appears to confer prognostic favor in PSC and a lower risk to develop adverse effects (1). Thus, it remains to be determined in prospective studies whether aGP2-positive CD patients characterized by complicated CD with fibrotic adverse effects also demonstrate severe PSC.

All 5 PSC patients with cholangiocarcinoma demonstrated aGP2_1_ and/or aGP2_4_ IgA. This might be a hint that aGP2 IgA occurs earlier in the disease course and that the tolerance break is linked with the mucosal microbiota interaction of GP2. In this context, the elevation of aGP2_3_ IgG in patients with CF is of interest since approximately 30% of patients with CF have clinically significant liver disease (46).

The finding that aGP2_1/4_ and aGP2_1/2/3/4_ IgA demonstrated significantly lower prevalences in PSC patients with CD was surprising. We speculate that PSC with concomitant CD might be different from CD without obvious liver involvement regarding the loss of tolerance to GP2. However, this notion needs to be treated with caution since it could be confirmed neither in the single cohorts nor by logistic regression analysis.

As most multi-center studies of this kind, our study lacks perfection. The controls were not age matched to patients with PSC due to the younger age of the controls with CF. Further, there was a high level of diversity in the distinct PSC cohorts of the four European hospitals regarding number of patients and their phenotype. The established independent predictors of cirrhosis aGP2_1_ and aGP2_4_ IgA could not be associated in all single cohorts with the concomitant occurrence of cirrhosis.

Altogether, combined aGP2_1_ and aGP2_4_ analysis is required for sensitive PSC-specific autoAb testing and should be preferred to the autoAb analysis against single aGP2 isoforms only. aGP2_1_ and aGP2_4_ IgA might be predictors of cirrhosis in PSC and, thus, an useful alternative for risk prediction. Elevated aGP2 IgA may demonstrate a link to fibrotic changes observed in IBD in particular in CD. Prospective studies including patients tested pre- and post-liver transplantation will provide excellent hints regarding the pathophysiological role of these autoAbs.

## Author contributions

DR and PS conceived of the study and participated in its design and data evaluation. RK and JS conducted the transduction experiments and participated in the development of the indirect fluorescence assay. MS participated in the development of the indirect fluorescence assay and carried out the assays. MP, TT, DCB, JP, MM, DB, JH, ML, KC, AF, and CS recruited patients, provided patient data and participated in the statistical evaluation thereof. All authors read and approved the final manuscript.

### Conflict of interest statement

DR is a shareholder and employee of GA Generic Assays GmbH and Medipan GmbH. MS receives a grant from GA Generic Assays GmbH. Both have no further personal or professional interests to declare.

The remaining authors declare that the research was conducted in the absence of any commercial or financial relationships that could be construed as a potential conflict of interest.

## Abbreviations

GP2: zymogen glycoprotein 2
PSC: primary sclerosing cholangitis
GP2_1_: GP2 isoform 1
IFA: indirect immunofluorescence assay
OR: odds ratio
CI: confidence interval
IBD: inflammatory bowel disease
CD: Crohn's disease
UC: ulcerative colitis
autoAb: autoantibody
ELISA: enzyme-linked immunosorbent assay
GPI: glycosylphosphatidylinositol
AIH: autoimmune hepatitis
CF: cystic fibrosis
HS: healthy subjects
IQR: interquartile range
LTx: liver transplantation
YI: Youden index
